# Non-tuberculous mycobacterial ocular infection masquerading as choroidal tumour – a diagnostic conundrum

**DOI:** 10.3205/oc000114

**Published:** 2019-07-05

**Authors:** Khy Ching Yeap, Premala Devi Sivagurunathan, Puspha Raman, Khairul Husnaini Mohd Khalid

**Affiliations:** 1 Department of Ophthalmology, Hospital Tuanku Ampuan Najihah Kuala Pilah, Malaysia

**Keywords:** non-tuberculous mycobacteria, atypical, Mycobacterium Fortuitum, choroidal tumour

## Abstract

**Purpose:** To report a rare case of non-tuberculous mycobacterial (NTM) choroiditis masquerading as choroidal tumour, where the initial diagnosis was masked by keratitis.

**Case** **description:** A 57-year-old heroin chaser with a pre-existing left eye blindness due to past blunt trauma presented with diffuse bacterial keratitis on the same side. Systemic examination revealed multiple non-tender cervical lymphadenopathies. B-scan ultrasonography showed a hyperechoic choroidal mass with surrounding exudative retinal detachment, resembling a choroidal tumour. However, computed tomography (CT) and magnetic resonance imaging (MRI) scan findings were suggestive of inflammatory choroidal changes. Inflammatory markers were significantly raised and infective screening was positive for HIV and Hepatitis C. Tuberculosis workup was normal. In view of intractable pain, evisceration was done and his vitreous humour was sent for polymerase chain reaction (PCR). It was reported to be positive for Mycobacterium Fortuitum.

**Conclusion:** NTM ocular infections are rare, challenging to diagnose, and potentially sight threatening. Early recognition and prompt treatment is life and vision saving.

## Introduction

Non-tuberculous mycobacteria (NTM) are ubiquitous organisms most commonly found in soil, water, air, and food. Although NTM ocular infections are rare, there has been a steady increase of such cases in the past two decades [1]. Risk factors for ocular NTM infections are trauma, corneal surgery or previous infection, corticosteroid use, and systemic immunosuppression [2]. The commonly isolated NTM species in ocular infections are the rapid growing types including Mycobacterium Chelonae, Mycobacterium Fortuitum and Mycobacterium Abscessus [3]. 

M. Fortuitum is usually associated with indolent keratitis and low grade ocular inflammation. The first case was reported in 1965 where M. Fortuitum caused chronic keratitis following corneal foreign body removal [4]. After that more cases of M. Fortuitum keratitis were reported in laser refractive surgery or keratoplasty patients [2], [5]. There are few reported cases of M. Fortuitum endophthalmitis after vitrectomy and glaucoma surgeries [6]. These cases are characteristically caused by an initial corneal trauma or surgery and followed by a chronic course of disease progression. We present a rare case of M. Fortuitum causing rapidly destructive keratitis associated with choroidal granuloma, leading to an unfavourable outcome in a HIV patient.

## Case description

**Figure 1:**
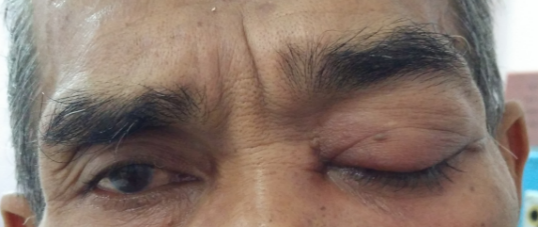
External photograph showing swollen periorbital tissue with proptosis

**Figure 2:**
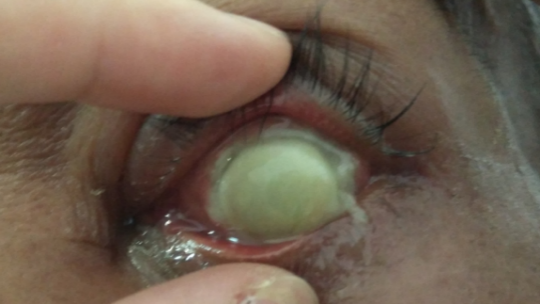
External photograph showing melting central corneal ulcer with hypopyon

**Figure 3:**
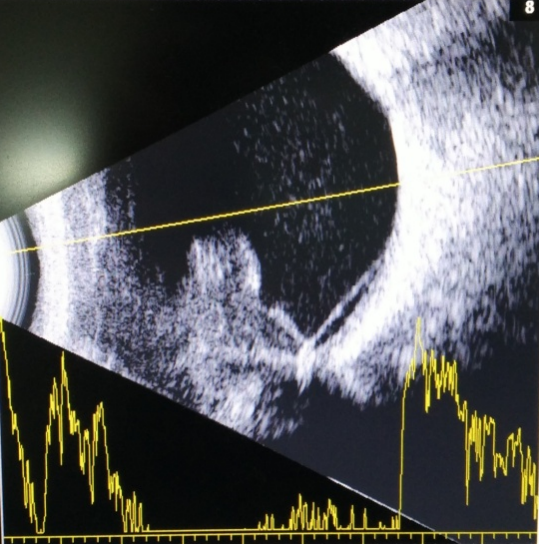
B-scan showing hyperechoic mass with surrounding exudative retinal detachment

**Figure 4:**
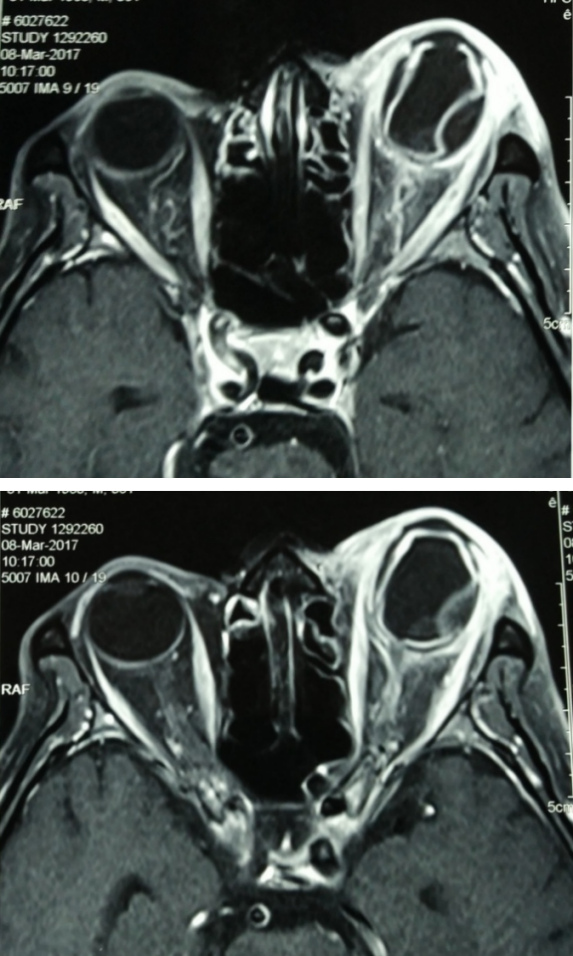
MRI orbit showing left choroidal detachment with lens displacement

A 57-year-old man, who has been blind in the left eye secondary to blunt trauma for the past ten years, presented with a three-day history of left eye pain associated with purulent discharge after a foreign body entry. The patient had no known medical illness and he was a heroin chaser. On examination, he had no light perception in the left eye with positive relative afferent pupillary light defect. There was a melting central corneal ulcer, associated with proptosis, periorbital swelling, diffuse conjunctival chemosis, and injection (Figure 1 (Fig. 1), Figure 2 (Fig. 2)). His intraocular pressure, measured gently with a tonopen, was elevated to 24 mmHg. The iris and fundus details were not visible due to the opaque cornea. B-scan ultrasonography showed a hyperechoic mass with surrounding exudative retinal detachment, resembling a choroidal tumour (Figure 3 (Fig. 3)). Systemic examination showed multiple non-tender cervical lymphadenopathies with rubbery consistency, measuring 0.5 cm by 0.5 cm. Otherwise, his cardiovascular, respiratory, and abdominal examinations were normal with no evidence of malignancy or systemic infection. The patient was initially diagnosed with left bacterial keratitis secondary to a foreign body entry with possibly an underlying choroidal tumour. Our differential diagnosis was left bacterial keratitis resulting from a ruptured bullous keratopathy secondary to chronically raised IOP due to the tumour. However, the MRI imaging showed left choroidal detachment with lens displacement, surrounding inflammatory changes and no evidence of tumour. There was no increase in vascularity within the mass as well (Figure 4 (Fig. 4)). Blood investigations showed a markedly elevated erythrocyte sedimentation rate (ESR) of 105 mm/hour (reference range: 1–10 mm/hour), raised C-reactive protein of 69.6 mg/L (reference range: <5 mg/dL) with positive HIV and Hepatitis C status. His corneal scraping grew Pseudomonas Aeruginosa. 

Due to the patient’s immunocompromised status, markedly elevated ESR, lymphadenopathy, and inflammatory changes of the mass, a possible diagnosis of intraocular tuberculosis (TB) was made. However, TB work-up, which included chest X-ray, Mantoux test and sputum to look for acid-fast bacilli were normal. The patient was started on Ceftazidime 5% and fortified Gentamicin 0.9% eye drops which was sensitive for the Pseudomonas Aeruginosa species and two IOP lowering agents. Despite the initial targeted treatment, the patient’s condition worsened. His cornea perforated within two days of presentation and the patient was in intractable pain, not responding to analgesics. After an elaborated discussion and counselling, we proceeded with evisceration of the affected eye. The corneal tissue and vitreous humour was sent for TB polymerase chain reaction (PCR). The PCR result came back positive for M. Fortuitum. The patient was not started on any systemic antibiotics as there was no evidence of systemic dissemination of the infection.

## Discussion

This case illustrates that NTM keratitis and choroiditis are rare opportunistic infections that can pose both diagnostic and therapeutic challenges. In the literature, the average duration from onset of symptoms to diagnosis is 10 weeks [3]. In this case, it took approximately 6 weeks from the onset of symptoms for the diagnosis of NTM choroiditis to be confirmed. From the systematic reviews and the reported cases, it is deducible that NTM infections are great masquerades. The clinical features and indolent nature of NTM keratitis can mimic those of infectious keratitis by other pathogens, especially fungus, herpes simplex virus, and Acanthamoeba [1]. An indolent inflammatory process and recalcitrance to traditional antibacterial therapy should increase the level of suspicion of NTM infections. Contrary to the reported cases, this patient presented with progressive keratitis that resulted in perforation despite the intensive medical treatment. The presence of a dual infection of Pseudomonas Aeruginosa and M. Fortuitum may explain the altered course of the disease.

The B-mode ultrasonography features of this patient mimicked a choroidal tumour with collar stud appearance with low internal reflectivity. Similar to our case, Lai et al. from Japan reported a case of NTM choroiditis in a HIV patient which was mistaken for ocular lymphoma [7]. All the other reported NTM choroiditis cases also experienced delay in diagnosis due to misdiagnosis, misidentification of the organism, and delay in taking cultures [1].

The three commonest NTM species, M. Chelonae, M. Fortuitum and M. Abscessus are part of Group IV NTM, which are also known as rapidly growing NTM. These organisms take 7–10 days to culture, unlike other groups of NTM, which take up to 2–4 weeks [2], [8]. Based on the literature, NTM are best diagnosed firstly using a Löwenstein-Jensen medium to culture for acid-fast bacilli. However, it was reported that the initial NTM isolates identified via conventional biochemical techniques could possibly belong to different NTM subtypes, hence PCR is employed to reduce misidentification of the species [9]. This is important as different NTM species have different antimicrobial susceptibilities.

Intraocular NTM infections were reported to have poorer response to medical treatment as compared periocular, adnexal, and ocular surface NTM infections. Studies have shown that Amikacin and Clarithromycin were more efficacious, while fluoroquinolones in general were less effective [3], [9]. Combination therapy using amikacin, a fluoroquinolone, and a macrolide is preferred over monotherapy to reduce resistance and to increase efficacy by increasing penetration through the corneal epithelium. Presence of a foreign body or implant and a non-responding intraocular NTM infection may warrant surgical intervention such as removal of the foreign body or implant itself, pars plana vitrectomy, evisceration, or enucleation. In this patient, evisceration was necessary to remove the infected ocular tissue. 

## Conclusion

NTM ocular infections are rare and pose diagnostic challenges. A delay in diagnosis leads to severe morbidity and poor visual outcome. Hence, high index of suspicion, prompt investigation and treatment could be sight saving. Therapeutic surgical intervention may be necessary to control the infection.

## Notes

### Competing interests

The authors declare that they have no competing interests.

### Patient consent

Written consent to publish the case report was obtained. 

### Acknowledgments

- Radiology Department of Hospital Tuanku Ampuan Najihah, Kuala
- Pilah for interpretation of relevant imaging
